# *SCN5A *allelic expression imbalance in African-Americans heterozygous for the common variant p.Ser1103Tyr

**DOI:** 10.1186/1471-2350-11-74

**Published:** 2010-05-14

**Authors:** Stacy AS Killen, Jennifer Kunic, Lily Wang, Adele Lewis, Bruce P Levy, Michael J Ackerman, Alfred L George

**Affiliations:** 1 Department of Pediatrics, Vanderbilt University, Nashville, TN, USA; 2 Department of Medicine, Vanderbilt University, Nashville, TN, USA; 3 Department of Biostatistics, Vanderbilt University, Nashville, TN, USA; 4 Tennessee Medical Examiner's Office, Nashville, TN, USA; 5 Department of Pediatrics, Mayo School of Medicine, Rochester, MN, USA

## Abstract

**Background:**

Heterozygous and homozygous carriers of *SCN5A*-p.Ser1103Tyr, a common genetic variant with functional effects among African-Americans, have an increased risk of sudden death. We hypothesized that some heterozygous carriers may have unequal expression of wild-type and variant alleles and secondarily that predominance of the variant gene copy could further increase risk for sudden death in this population.

**Methods:**

We quantified allele-specific expression of *SCN5A*-p.Ser1103Tyr by real-time reverse-transcription polymerase chain reaction (RT-PCR) in heart tissue from heterozygous African-American infants, who died from sudden infant death syndrome (SIDS) or from other causes, to test for allelic expression imbalance.

**Results:**

We observed significant allelic expression imbalance in 13 of 26 (50%) African-American infant hearts heterozygous for *SCN5A*-p.Ser1103Tyr, and a significant (p < 0.0001) bimodal distribution of log_2 _allelic expression ratios. However, **t**here were no significant differences in the mean log_2 _allelic expression ratios in hearts of infants dying from SIDS as compared to infants dying from other causes and no significant difference in the proportion of cases with greater expression of the variant allele.

**Conclusions:**

Our data provide evidence that *SCN5A *allelic expression imbalance occurs in African-Americans heterozygous for p.Ser1103Tyr, but this phenomenon alone does not appear to be a marker for risk of SIDS.

## Background

Every year in the United States more than 300,000 people die suddenly from an unexpected cardiac event [1], with coronary artery disease accounting for 80% of sudden cardiac death (SCD) in the adult population [2]. However, in children and adolescents, inherited arrhythmias, such as long-QT syndrome, Brugada syndrome, familial atrial fibrillation, catecholaminergic polymorphic ventricular tachycardia, and Wolff-Parkinson-White syndrome, are a leading cause of sudden death [2]. Sudden death from cardiac causes is more common in African-Americans than in Caucasians [3,4]. In adults, sudden death from nonatherosclerotic heart disease is more frequent in African-Americans, while during infancy, African-Americans are twice as likely as Caucasians to die from sudden infant death syndrome (SIDS) [5].

Mutations in *SCN5A*, the gene encoding the pore-forming α-subunit of the cardiac voltage-gated sodium channel, have been associated with sudden death in long QT syndrome and Brugada syndrome [6]. A common *SCN5A *genetic variant (p.Ser1103Tyr) that is found in 13% of African-Americans is associated with an increased risk for cardiac arrhythmia and sudden cardiac death in that population [7,8]. Further, the p.Ser1103Tyr variant is over-represented in African-American SIDS victims as compared to non-SIDS infant death cases [9,10] and sodium channels with the variant allele exhibit functional defects that may increase risk for life-threatening cardiac arrhythmias [7]. Because infants dying suddenly and unexpectedly are usually brought to autopsy, they provide a unique opportunity for studying tissue-specific expression of a potentially lethal genetic variant.

Allelic variation in gene expression is a well-recognized phenomenon [11,12]. We hypothesized that African-Americans heterozygous for *SCN5A*-p.Ser1103Tyr exhibit unequal expression of wild-type and variant alleles (allelic expression imbalance) and that predominance of the variant gene copy might further increase the risk for sudden death in this population.

## Methods

### Tissue samples

De-identified, frozen, postmortem heart tissues from African-American SIDS cases and African-American infant controls were obtained from two sources: the Tennessee Medical Examiner (29 cases, 16 controls), and a previously reported cohort collected at Mayo Clinic (10 cases, 30 controls) [10]. The SIDS cases from the Tennessee Medical Examiner met a rigorous definition of SIDS [13] or sudden unexpected infant death (SUID) [14]. Use of anonymous human tissues in this study was approved by the Institutional Review Board of Vanderbilt University.

### RNA extraction, cDNA synthesis, and genotyping

Total RNA was extracted from pulverized heart muscle using TRIzol reagent (Invitrogen, Carlsbad, CA) according to the reagent supplier's instructions, followed by DNase digestion and final purification with RNeasy MinElute (Qiagen, Valencia, CA). RNA quality was determined by spectrophotometry and gel electrophoresis, and samples exhibiting degradation were not used. RNA samples were diluted to a concentration of 1 μg/μl prior to performing reverse transcription (RT) to synthesize cDNA used for genotyping and for allele-specific mRNA measurements. Reverse transcription was performed using 3 μg RNA, 3.75 μM random hexamers (Applied Biosystems, Foster City, CA), 0.5 mM deoxynucleoside triphosphates (Roche, Basel, Switzerland), 1× M-MLV buffer (Promega, Madison, WI), 10 mM DTT, 40 units RNase inhibitor (Promega), and 200 units M-MLV reverse transcriptase (Promega). Reverse-transcription reactions were incubated sequentially for 10 minutes at 25°C, 50 minutes at 42°C, and 15 minutes at 70°C. Genotyping for the *SCN5A*-p.Ser1103Tyr (c.3308C>A) variant was performed by direct sequencing of a 230 bp amplicon (nucleotides 3217-3446 of the open reading frame of Genbank: NM_198056) generated by RT-PCR (primers: forward, TCCAGCAAGCAGCAGGAATC; reverse, TCAGCGGTGTTGGTCATGTC) from each RNA sample. The *SCN5A*-p.Ser1103Tyr variant has also been designated as p.Ser1102Tyr based on the amino acid sequence of an alternatively spliced transcript (Genbank: NM_000335). To be consistent with the numbering scheme used widely for *SCN5A *mutations, we used p.Ser1103Tyr based upon the canonical transcript.

### Allele-specific quantitative RT-PCR

Heterozygous samples were evaluated for *SCN5A *allelic expression imbalance using real-time quantitative RT-PCR and allele-specific TaqMan probes that contained a minor groove binder (MGB) at the 3' end (p.Ser1103: FAM-ACTGCCTCCTCTGA-MGB; p.Tyr1103: FAM-ACTGCCTACTCTGA-MGB; allele-specific sequences are underlined) to quantify the separate alleles. Amplification primers (forward: CCCTCCGGATTCCAGGAC; reverse: CTGCCCTCGGAGCAACTG) used in the assay generated a 156 bp amplicon (nucleotides 3264-3419 based on the open reading frame of Genbank: NM_198056) with a two-temperature cycling protocol (95°C for 20 s followed by 45 cycles of 95°C, 1 s and 60°C, 20 s). Under these conditions neither TaqMan probe exhibited cross-reactivity with the opposite allele. Amplicons corresponding to each allele were cloned into a plasmid vector and used as cDNA standards. All reactions were performed in triplicate on a 7900HT Fast Real-Time PCR system (Applied Biosystems). Individual reactions (20 μl) contained 1× Fast Universal PCR Master Mix (Applied Biosystems), forward and reverse primers (3 μM), an allele-specific probe (10 μM), and 5 μl of cDNA (or 5 μl water for template-negative controls). The ability of the assay to quantitatively distinguish different proportions of the two alleles with high specificity was assessed using plasmid cDNA standards in the following mass ratios (p.Tyr1103:p.Ser1103): 4:1, 2:1, 1:1, 1:2, and 1:4. The cDNA standards were diluted to a final concentration of 0.001 ng/μl after preliminary studies determined that this concentration produced cycle threshold values similar to heart tissue RNA. Assays were performed in triplicate and each RNA sample was assayed from 2-3 independent preparations of cDNA.

### Data analysis

Initial data analysis was performed using Applied Biosystems Sequence Detection System software (version 2.2.2). Two principles guided our approach to deduce allelic expression ratios. First, the cycle threshold (C_T_) is inversely proportional to the copy number of the template, assuming 100% amplification efficiency, and second, there is a linear relationship between log_2 _of the allele ratio and ΔC_T _(difference between C_T _values for each allele). A standard curve generated from assaying cDNA standards mixed at known ratios and fitted with the equation log_2_(p.Tyr1103:p.Ser1103) = αΔC_T _+ β (α and β are fit parameters) was used to interpolate the ratio of allele expression for case and control samples.

Statistical analysis was conducted using SAS (version 9.1.3, SAS institute, Cary, NC). Because multiple RT-PCR allelic imbalance assays were performed for each case and control, it was important to account for dependencies from repeated observations from the same subject. To this end, we used a mixed effects model to assess the allele ratios, with log_2_(p.Tyr1103:p.Ser1103) as the outcome variable, with fixed effects, including sample source (Tennessee ME versus Mayo Clinic samples), age, gender, group (cases vs. controls), and random effects, including batch, subject and assay plate. The random batch effects were created based on unique combinations of PCR date and assay date. To obtain the estimates of Best Linear Unbiased Prediction (BLUP) of allelic expression imbalance for each subject, we constructed linear combinations of estimated parameters from this mixed effect model for each subject. Allelic imbalance was defined by log_2_(p.Tyr1103:p.Ser1103) ratio significantly different from zero. The null hypothesis, log_2_(p.Tyr1103:p.Ser1103) = 0, describes the absence of allelic imbalance. A Bonferroni-corrected p-value of p < 0.002 was considered statistically significant. We also plotted the p.Tyr1103:p.Ser1103 log_2 _allelic expression ratios for all cases and controls as a function of the count and used the Gaussian Fit option in OriginPro7 (OriginLab Corp., Northhampton, MA) to estimate the smoothed density function of the mean log_2 _allelic expression ratios. We then compared the goodness of fit of a two component mixture model and a normal model to the data using a likelihood ratio test.

## Results

We identified 17 African-American SIDS cases and 9 African-American non-SIDS infant death controls that were heterozygous for *SCN5A*-p.Ser1103Tyr and that had available frozen heart tissue from which we were able to isolate intact, high quality total RNA. We developed a quantitative assay to measure allele-specific expression in heart tissue from African-American infants heterozygous for *SCN5A*-p.Ser1103Tyr using allele-specific real-time reverse-transcription PCR (RT-PCR). Allelic imbalance assays performed on monoallelic samples demonstrated negligible allele cross-reactivity.

**Figure 1:**
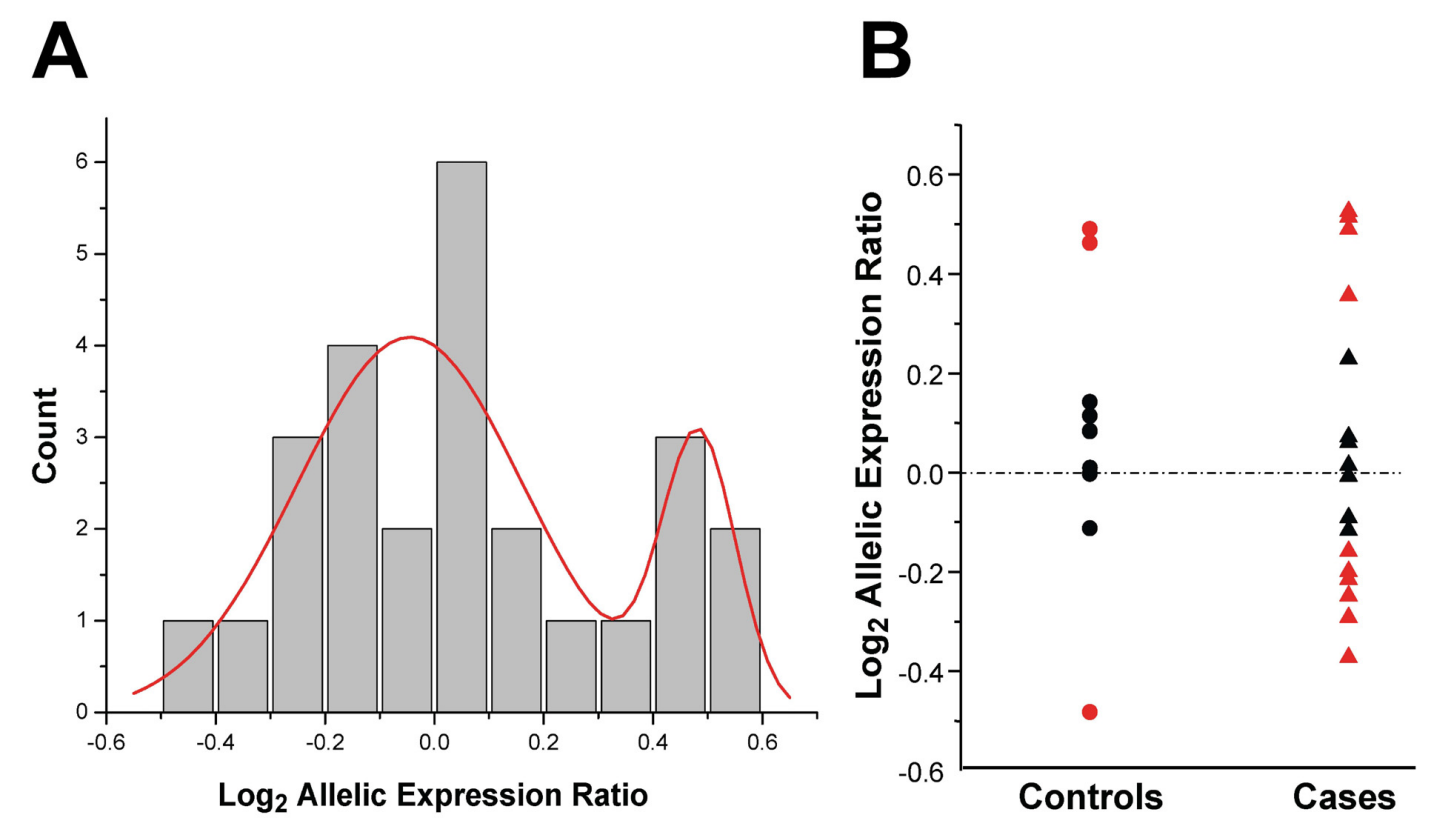
**Allelic variation in cardiac SCN5A expression in African-American infants**. (A) The log_2 _p.Tyr1103:p.Ser1103 allelic expression ratios for all cases and controls are plotted as a function of the count and fitted with Gaussian functions (red line). The goodness of fit for a two component model was significantly better than a single mode (p < 0.0001), thus demonstrating a bimodal distribution of allelic expression ratios. (B) Distribution of log_2 _allelic expression ratios for SIDS cases and controls. Red colored symbols represent subjects with log_2 _allelic expression ratios significantly different from zero using a Bonferroni corrected level of significance (p < 0.002).

Analysis of all subjects revealed that 13 of 26 (50%) African-American heterozygous infant hearts exhibited log_2 _allelic expression ratios significantly different from zero (p < 0.002), indicating the presence of allelic expression imbalance. Further, we observed a significant (p < 0.0001) bimodal distribution of log_2 _allelic expression ratios (Fig. 1A). These data indicate that a subset of African-American infants who are heterozygous for p.Ser1103Tyr have unequal cardiac expression of the common and variant alleles. In individuals subjects, allelic expression imbalance was present in 10 of 17 cases (59%) and 3 of 9 controls (33%; Fig. 1B). However, analysis of allelic expression data using a mixed effects model demonstrated that the log_2 _p.Tyr1103:p.Ser1103 expression ratios for SIDS cases was not significantly different from that of the controls. Further, the proportion of cases with elevated variant allele expression was not statistically different from controls (Fisher's exact test, p > 0.8). Therefore, although our data provide significant evidence of *SCN5A *allelic expression imbalance in heterozygous African-American hearts, there is no evidence for an association with the risk of SIDS.

## Discussion

Inherited arrhythmias are a leading cause of sudden cardiac death in infants, children, and adolescents [2]. African-American infants have a two-fold higher risk of sudden death as compared with Caucasian infants [5]. One potential explanation of sudden death risk in African-Americans comes from work on the common genetic variant *SCN5A-*p.Ser1103Tyr. Thirteen percent of African-Americans carry at least one copy of the *SCN5A-*p.Tyr1103 missense variant, for which *in vitro *evidence supports having a potential pathophysiologic role in increased cardiac arrhythmia risk [7]. Plant and colleagues demonstrated an over-representation of homozygous *SCN5A-*p.Tyr1103 subjects in a cohort of 133 African-American SIDS victims [9]. This study further demonstrated that *SCN5A*-p.Tyr1103 channels exhibit increased persistent sodium current (an arrhythmia susceptibility mechanism observed in LQTS) particularly when exposed to intracellular acidosis. A subsequent study demonstrated a significantly higher prevalence of heterozygous *SCN5A*-p.Ser1103Tyr carriers among 71 African-American cases of sudden unexplained infant deaths as compared with African-American controls [10].

We hypothesized that some African-Americans heterozygous for *SCN5A*-p.Ser1103Tyr have allelic expression imbalance with predominance of the variant gene copy. In this situation, greater relative expression of the minor allele (p.Tyr1103) might contribute more to arrhythmia susceptibility and further increase the risk of sudden cardiac death. Our data suggest that allelic expression imbalance occurs with *SCN5A *but we did not observe a significant difference in allelic expression ratios or the proportion of cases with elevated variant expression. One potential limitation of our study stems from alternative splicing of *SCN5A *mRNA. Our assay conditions do not distinguish expression of the two alleles present on differentially spliced *SCN5A *transcripts and therefore we can not exclude that allelic expression imbalance involves a specific splice variant.

Many autosomal genes can exhibit multi-fold differences in allele-specific expression even in healthy tissues [11,12]. In some settings, such as breast cancer associated with *BRCA1 *or *BRCA2 *mutations, allelic expression imbalance may underlie increased disease risk [15]. The most likely explanation for this phenomenon is the presence of *cis*-regulatory polymorphisms [16]. A prior study identified a common *SCN5A *haplotype in Asians that was associated with significantly reduced promoter activity measured *in vitro *and correlated with variability in PR interval, QRS duration, and QRS widening during exposure to sodium channel blocking drugs [17]. This particular variant haplotype was not present in African-Americans, and the study did not examine allelic variation in cardiac *SCN5A *expression. But this work did establish a precedent for predicting that allelic expression imbalance in *SCN5A *may occur and may have potential clinical consequences.

## Conclusions

In conclusion, our study demonstrated that allelic variation in cardiac *SCN5A *expression occurs in African-Americans heterozygous for p.Ser1103Tyr. This finding is biologically important because it demonstrates that *SCN5A *gene expression can exhibit allelic imbalance. However, this phenomenon alone was not associated with risk for SIDS in the cohorts we examined. Allelic expression imbalance in *SCN5A *could be one mechanism among a host of genetic, epigenetic, and environmental phenomena that together modify sudden death risk in other susceptible hosts.

## Competing interests

The authors declare that they have no competing interests.

## Authors' contributions

SASK designed and performed experiments, analyzed data and wrote the manuscript. JK assisted with experiments and data analysis. LW contributed to statistical analysis of data. AL, BL and MJA procured tissue samples. ALG conceived of the study, helped plan experiments, analyzed data and wrote the manuscript. All authors have read and approved the final manuscript.

## Pre-publication history

The pre-publication history for this paper can be accessed here:

http://www.biomedcentral.com/1471-2350/11/74/prepub
